# Supplementary material for: Precision Editing as a Therapeutic Approach for β-Hemoglobinopathies
Source: Int J Mol Sci. 2023 May 31;24(11):9527. doi: 10.3390/ijms24119527 (PMC10253463; doi:10.3390/ijms24119527)
Supplement: Supplementary file 1 [file ijms-24-09527-s001.zip › ijms-2386476-supplementary.pdf]

Clinical Trial Identifier	***Sponsors***	***Genome editor***	***Enrolled/enrolling patients***	***Delivery Mode***	***Genomic Target***	***References***
NCT03432364; ST-400-01	Sangamo Therapeutics and Sanofi	Zinc Finger Nucleases (ZNF)	Sickle cell disease Transfusion dependent β - thalassemia	mRNA electroporation	Erythroid enhancer of *BCL11A* gene	[170]
NCT03655678; CTX001-111	VERTEX Therapeutics and CRISPR Therapeutics	CRISPR-Cas9	Transfusion dependent β-thalassemia	RNP electroporation	Erythroid enhancer of *BCL11A* gene	[173,172]
NCT03745287; CTX001-121	VERTEX Therapeutics and CRISPR Therapeutics	CRISPR-Cas9	Sickle cell disease	RNP electroporation	Erythroid enhancer of *BCL11A* gene	[171]
NCT04211480	Bioray Laboratories Xiangya Hospital of Central South University PLA 923 Hospital	CRISPR-Cas9	β0/β0 and β+/β+ thalassemia	RNP electroporation	BCL11A binding site in *HBG* promoter	[136]
NCT04819841; GPH101-001; CEDAR	Graphite Bio, Inc	CRISPR-Cas9	Sickle cell disease	Not disclosed	*HBB* gene	[173]
NCT04853576; EM-SCD-301-001	Editas Medicine, Inc	CRISPR-Cas9	Sickle cell disease	RNP electroporation	*HBG1/2* promoter	[173]

Table S1. Gene-editing clinical trials for beta-thalassemia and sickle cell disease.

Genetic disease	*Genomic Locus*	*Mutation*	*Genome editing tool*	*Results*	*References*
Sickle cell disease	*HBB* exon 1	SCD point mutation correction	ZFN, CRISPR-Cas9,	HbA production	[26,94,96]
Sickle cell disease	*HBG1/2* promoter	Disruption of LRF biding site or recruitment of KLF1	ABE	HbF reactivation	[140]
Sickle cell disease	*HBG1/2* promoter	Deletion of a 13.6 kb genomic region involving β- and δ-globin genes	CRISPR-Cas9	HbF reactivation	[107,109]
Sickle cell disease	*BCL11A* erythroid enhancer	Disruption of the GATA1 binding site	CRISPR-Cas9	HbF reactivation	[135,136]
Sickle cell disease	*HBG1/2* promoter	Disruption of the ZBTB7A binding site within the *HBG1/HBG2* promoter	CRISPR-Cas9	HbF reactivation	[144]
Sickle cell disease	*KLF1*	Disruption of *KLF1*	CRISPR-Cas9	HbF reactivation	[149]
Sickle cell disease	*PPP6C*	Disruption of *PPP6C*	CRISPR-Cas9	HbF reactivation	[169]
Beta thalassemia	*HBB* exon 2	Correction of the CD41/42ΔTTTC mutation	CRISPR-Cas9	HbA production	[97]
Beta thalassemia	*HBB* intron 2	Correction of the *HBB* IVS2-654 mutation	CRISPR-Cas9, TALENs	Splicing correction, HbA production	[99,100]
Beta thalassemia	*HBB* intron 1	Correction of the IVS1-110G>A mutation	CRISPR-Cas9, TALENs, ABE	Splicing correction, HbA production	[103,104,112]
Beta thalassemia	*HBG1/2* promoter	Disruption of regulating elements	TALENs	HbF reactivation	[25]
Beta thalassemia	*HBG1/2* promoter	13kb deletion (Sicilian HPFH mutation)	CRISPR-Cas9	HbF reactivation	[106]

Beta thalassemia	*HBG1/2* promoter	Introduction of HPFH mutations at -115C or -114C	CBE	HbF reactivation	[111]
Beta thalassemia	*HBG1/2* promoter	Introduction of HPFH mutations at -113A	ABE	HbF reactivation	[113]
Beta thalassemia	*BCL11A* erythroid enhancer	Disruption of the *BCL11A* erythroid enhancer	ZNF	HbF reactivation	[27]
Beta thalassemia	*HBB*	Correction of β039-globin mutations	CRISPR-Cas9	HbA production	[105]
Beta thalassemia	*HBG1/2* promoter and *BCL11A* erythroid enhancer	Simultaneous disruption of the *BCL11A* erythroid enhancer with the LRF/BCL11A binding sites on the *HBG1/2* promoter	CRISPR-Cas9	HbF reactivation	[138]
Beta thalassemia and Sickle cell disease	*BCL11A* erythroid enhancer	Introduction of a point mutation on the +58-DHS of the *BCL11A* erythroid enhancer	CBE	HbF reactivation	[139]
Beta thalassemia	*BCL11A* erythroid enhancer and *HBB* promoter	Simultaneous disruption of the *BCL11A* erythroid enhancer and correction of the *HBB* -28A>G promoter mutation	CBE	HbF reactivation	[139]

Table S2. Gene editing strategies for beta-hemoglobinopathies applied in patient derived cells.
